# Patient-initiated follow-up of early endometrial cancer: a potential to improve post-treatment cardiovascular risk?

**DOI:** 10.1007/s00404-021-06166-9

**Published:** 2021-08-07

**Authors:** Racheal Louise Johnson, Cheng Choy

**Affiliations:** 1 St James University Teaching Hospital, Leeds Teaching Hospital, 9 Gledhow Park Drive, Beckett Street, Leeds, LS7 4JT UK; 2 Huddersfield and Calderdale Teaching NHS Trust, Huddersfield, UK; 3 Calderdale Royal Hospital, Halifax, HX3 0PW UK

**Keywords:** Patient-initiated follow-up, Endometrial cancer, Weight loss, Cost analysis

## Abstract

**Purpose:**

Is patient-initiated follow-up, post-surgical treatment of early endometrial cancer safe and can it be used holistically to improve cardiovascular health? What are the cost implications of this model of follow-up?

**Methods:**

Retrospective data of 98 patients discharged to patient-initiated scheme since 2012. Service evaluation by anonymous patient feedback including physical health effects of the programme including weight loss. Financial cost was compared to traditional hospital-based follow-up over five years.

**Results:**

No evidence of recurrence over 54 months median follow-up in low-risk endometrioid endometrial cancer. Patient feedback indicates that the exercise course helped women reduce their BMI. Over one third women felt happier and one fifth felt more confident and had a better ability to cope with stress. Total of 91% patients would recommend this model of follow-up to friends or family in the same circumstance. European Society for Medical Oncology guidance suggests the number of hospital-based follow-up appointments required for this cohort would cost £109,760. Calculations in this paper examine the cost of patient-initiated follow-up and reflect an overall saving of around 96.5%.

**Conclusion:**

This service evaluation supports the claim that patient-initiated follow-up represents a safe alternative to the traditional hospital-based protocol. There is a potential for additional services to be offered to encourage and promote a healthy lifestyle linked to improving quality of life and cardiovascular survival following surgery for endometrial cancer.

**Implications for cancer survivors:**

Cardiovascular morbidity is the most common cause of death in endometrial cancer survivors. Incorporating an exercise course as part of routine follow-up can help reduce this risk. The friendships formed by this communal follow-up can contribute towards emotional health and recovery. This holistic approach should be incorporated into novel follow-up strategies to help reduce patient BMI and reduce cardiovascular risk.

## Introduction

The rising incidence of endometrial cancer is indicative of the global obesity pandemic: 40% of endometrial cancer cases are directly related to obesity and are therefore preventable [1]. Most patients present with early-stage disease confined to the uterus and up to 95% survive over 5 years [2]. There are more women surviving endometrial cancer than ever before. Between 2018 and 2019 there were 229,345 gynaecology-oncology outpatient hospital follow-up appointments in England compared to only 10,000 during 2005–2006 [3]. This poses an increasing demand on health care resources. European Society for Medical Oncology guidelines suggest endometrial carcinoma follow-up is based on clinician evaluations combining history, physical and gynaecological examination every 3–4 months for the first two years, then at six-month intervals until four years and a final annual check in the fifth year, [4] the focus being on detecting early recurrence.

Patients with International Federation of Gynaecology and Obstetrics (FIGO) stage one disease have a low risk of recurrence. There is no evidence that regular hospital-based follow-up post primary treatment can improve survival rates [5–8].Detection of asymptomatic recurrence with diagnostic technology has poor sensitivity and specificity and no survival benefit [5]. Over 97% of early endometrial cancer recurrences are symptomatic, with over 65% presenting with vaginal bleeding, a sign for which patients invariably seek prompt attention [9]. Alternative strategies to current hospital-based follow-up are being trialled, such as nurse-led telephone models. This would provide a less resource intense programme, having a positive effect on sustainability of the workforce, as well as being incredibly cost effective.

This retrospective review of 98 patients who underwent post primary surgical treatment of early endometrioid endometrial cancer between 2012 and 2018. They were discharged after one post-operative consultation, which assessed surgical recovery, reviewed histology and determined the need for adjuvant treatment. Those willing were subsequently enrolled on an educational day called ‘Next Steps’; a structured course educating women about their cancer, treatment and how to report recurrence symptoms. Next Steps also explored topics such as diet, exercise and smoking cessation. This information was provided by a gynaecology-oncology consultant, a cancer nurse specialist and a health instructor supplied by the local council. Patients were educated to look out for red flag symptoms such as vaginal bleeding, new discharge, unintentional weight loss or abdominal pain that would trigger an urgent outpatient review. Patients were not enrolled on the Next Steps programme if they required active or maintenance treatment or were otherwise limited in their capacity to participate in educational activities and self-initiated follow-up.

There was opportunity at the end of the Next Steps programme, to enrol on a free 10-week course titled ‘Upbeat CARES (Cancer Rehabilitation Exercise Service)’ exclusively for cancer survivors. This comprised a weekly 1 h exercise class, aiming to reduce body mass index (BMI) and improve overall cardiovascular health. The exercise class was tailored to provide a personalized exercise programme, specific to each patient’s needs and ran alongside a health tutorial which educated patients on the benefits of physical activity, healthy eating, alcohol and smoking cessation, as well as stress management. During the 10 week course patients had free access to additional activities at the Council Leisure Centre, such as use of the public swimming pool. After this 10-week period they could apply for two-year access at a reduced rate. Upbeat is funded and commissioned by the local Public Health department and managed by the Local Authorities Health Team.

## Methods

Our inclusion criteria were endometrioid endometrial cancer survivors with low risk of recurrence. This included all patients with stage 1a or 1b with grade 1 or 2 histology, under 50% myometrial invasion and no evidence of lymphovascular space invasion. Each had completed primary surgical treatment and had attended one outpatient consultation 6–12 weeks post-operatively and subsequently consented to attending the Next Steps course.

Data was collected retrospectively using patient notes. The primary patient outcome investigated was cancer recurrence. Our data also included patient demographics, an assessment of the self-initiated telephone consultation and patient evaluation of the education day and the Next Steps service.

Patient outcomes of the service were measured using a 28- item questionnaire sent to patients with an accompanying pre-paid return envelope. The questionnaire looked at patient satisfaction with the service and the information provided. This included asking direct questions such as if they would advise family/friends in similar circumstances to participate in the programme, as well as open questions on how the service could be improved. The feedback was coded by three main themes: generically positive, generically negative and service improvement suggestions. It also featured a 16-point evaluation of physical health-related determinants for those who completed the Upbeat health and exercise programme. A total of 47 out of 98 patients completed all parts of the questionnaire.

Approval was obtained from the Research Department of the clinical centre involved.

## Results

**Fig. 1:**
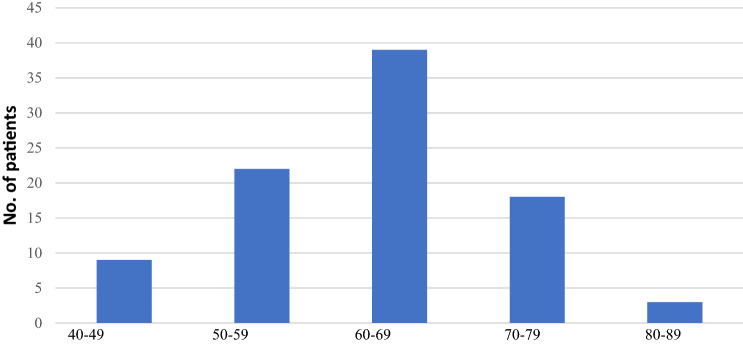
Age at time of primary surgery

**Fig. 2:**
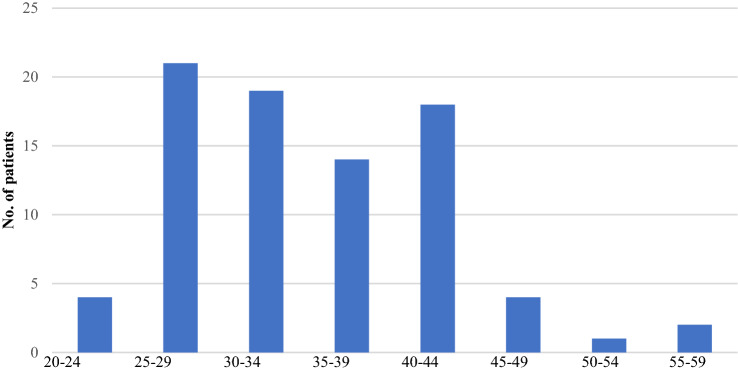
Body mass index at time of primary surgery

The median age in this cohort of patients was 62.8-year-old, ranging from 41 to 83 years old. See Fig. 1. The median body mass index was 34.29; class 1 obesity. This this ranged from 21 to 58.09. See Fig. 2.

**Fig. 3:**
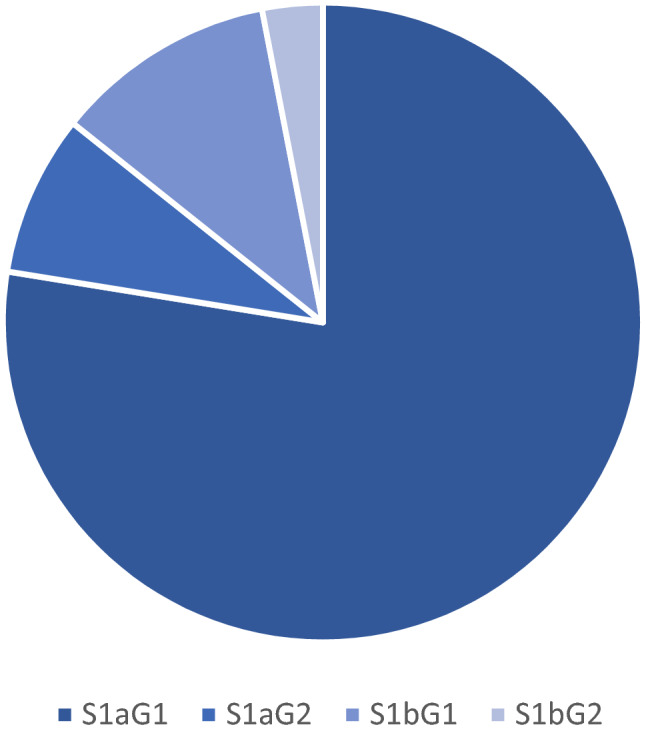
International federation of gynaecology and obstetrics staging endometroid endometrial cancer on final histology

All patients had endometroid type endometrial carcinoma. There were seventy-six patients’ stage 1a grade 1 on final histology, eight patients with stage 1a grade 2, eleven patients with stage 1b grade 1 and three patients with stage 1b grade 2. They were all treated with primary surgery and did not require any further treatment. See Fig. 3.

Median follow-up was 54 months. Progression free survival for the entire cohort was 97% (*n* = 3/98). Progression free survival was 100% in the stage 1a grade 1 (*n* = 0/76) cohort, 87.5% in the 1a grade 2 cohort (*n* = 1/8) and 85.7% in the 1b cohort (*n* = 2/14). All presented with vaginal bleeding, had local recurrence and were treated with radiotherapy. One of these patients unfortunately died from her disease, making the overall survival for the entire cohort 99%.

Nearly one fifth of patients (20/98) accessed the patient-initiated telephone service to discuss symptoms. This generated eleven hospital-based consultant clinic reviews. Only six of these referrals were ‘red flag’ symptoms including vaginal bleeding, discharge and pelvic pain. All three patients who developed recurrence were seen in clinic. The remaining five referrals who were since in clinic related to hormone replacement therapy and uro-gynecological complaints. This reflects patient’s engaging with self-initiated follow-up and using the service appropriately.

The other calls were triaged by specialist nurses and deemed not to require specialist gynaecology-oncology review such as enquires about vaginal dryness and clarity regarding the need for future cervical smear tests. Six of the calls were documented as not being related to their previous cancer or treatment, such as how to organize B12 injections prescribed by the general practitioner, but this is a relatively small number. There was one call to seek emotional support for general anxieties related to her previous cancer. All of these calls were managed appropriately.

An encouraging 91% of women felt like the information they received from the education day helped them understand their condition and treatment. The majority (43/47) of women provided positive written feedback. Recurring themes were the positive emotional value of meeting other women who had been through the same experience and feeling more informed about their circumstances.

Examples of positive feedback.

“*It was a good opportunity to learn more about the surgery and its implications and to meet up with a group of women who had experiences exactly what I had”*

*“Meeting others in the same situation and being able to talk to someone for support*”

The only negative feedback was from two patients who did not like seeing pictures of a medical nature (pictures of the uterus and operation were used) however one patient had wanted more medical detail to be shared. Two patients thought the course was too long. Overall 96% would recommend the day to family or friends in similar circumstances.

The vast majority (89%) of women felt confident to access services, if required, once they were discharged;

*“It helped me feel empowered rather than still ‘in the system’ “*

*“I was always given a clear idea of what happens next and when”*

*“All problems have been addressed and dealt with as rapidly as possible”*

Four women expressed negative concern about the lack of hospital follow-up;

*“I was surprised that there was no follow-up from the hospital to check all was well”*

*“I did feel a bit uneasy because I wasn’t having further check-ups. For my personality and others similar to me, I feel one check-up maybe 9months/1 year after the operation together with this course would have been better”*

*“It was a bit scary that there was no further follow-up, although everything is fine”*

*“It was a bit disconcerting not to have regular check-ups (as used to be the case) after surgery. But with hindsight, probably less stressful not to have hospital visits!”*

There were an additional two patients who both described their barriers to calling the hospital for advice as feeling like a ‘nuisance’. Both these women also commented that a way to improve the service would be for the hospital to initiate a telephone call to alleviate anxieties about calling themselves.

*“I still felt like I'd be bothering (being a nuisance) if I asked for help. (This is probably my personality).”*

*“I feel it would be beneficial if the cancer nurse team or similar were to ring people up. I am reasonably confident but felt at times worried to call anyone. So, a follow-up phone call after surgery would be beneficial.”*

A total of 32 out of 47 (68%) patients enrolled on the voluntary 10-week health course. Reasons why people did not join included the course being inconvenient, with a total of five patients saying it was too far or not at times to fit in with working hours. There were four patients who did not think the course was appropriate for them as they were already healthy and engaging with regular exercise. There were three who were prevented due to circumstance such as being a carer for her husband, finishing a university project and unfortunately one lady said she never received the letter to inform her of the course. Of those who attended 81% would recommend it to a friend/family in similar circumstances. Examples of positive feedback;

*“Started me on the way back to being fit and helped me feel better”*

*“The comradeship. The fact that others had been in the same situation. She (course instructor) was an amazing person helping everyone to achieve their goal”*

*“It is now 6 years since I joined Upbeat Group. We made new friends with whom we are still in regular contact. The dietary booklet and the exercises classes were excellent”*

*“Having lost 7 stone after the surgery I felt much better”*

Themes of negative feedback included access to the course, some people found public transport inconvenient, and it took one lady an hour to drive to the leisure centre. Two patients said the exercise was too *‘hard’* and they felt *‘shattered’* after and another commented on the inconsistency of staff leading the course. Patients also commented on the length of the course, suggesting six months may have been a more appropriate duration.

**Fig. 4:**
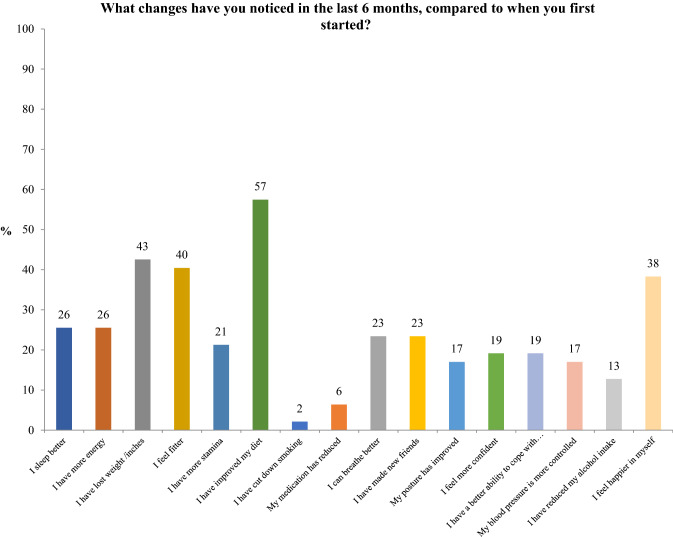
Physical health assessment questionnaire

Part of the questionnaire included an assessment of health improvements using sixteen parameters. 72% of patients felt their overall quality of life had improved after completing the course. 43% confirmed they had lost weight and 57% said they had improved their diet. Over one third women felt happier and one fifth felt more confident and had a better ability to cope with stress as a result of the course. See Fig. 4.

General comments and feedback;

*“I loved this course and I am so grateful to Calderdale Hospital for arranging it. I wish more people would have attended because it was life changing for me. I learnt so much, it was brilliant, Thanks.”*

*“The sessions I attended were so reassuring and it helped so much with my emotional recovery”*

## Discussion

Inactivity and high energy intake promote obesity, which is an independent risk factor for the development of endometrial cancer. Obesity is associated with increased risk of surgical complications and poorer survival rates compared to non-obese women [10]. Regular physical activity encourages weight loss and therefore reduces the risk of developing endometrial cancer. It improves cardiovascular morbidity and mortality, which remains the most common cause of death in endometrial cancer survivors [11, 12]. Most patients are motivated to engage with a change in lifestyle within the first six months following a cancer diagnosis [13].Thereby encouraging survivors to engage with weight loss services and physical activity is most likely to be successful in this window. This is reflected in this data as nearly half (43%) of patients reported weight loss after the Upbeat course.

Social and emotional support after surviving cancer can improve quality of life and protect against depression [14]. One quarter of women made new friends and one fifth felt they had improved their ability to cope with stress and improved their confidence as a result of the Upbeat course. Assessing patients individually in a clinical environment, does not provide an opportunity to cultivate this emotional benefit.

Feedback regarding apprehension to being discharged to patient-initiated follow-up, could represent the fear of cancer recurrence or the daunting responsibility of self-reporting symptoms. A recent randomized controlled trial demonstrated that traditional hospital-based follow-up reduced the ‘fear of recurrence’ compared to those with self-initiated follow-up, and that contributed to quality of life. It concluded that the reassurance of medical examination outweighed the anxiety before examinations [15]. However, this was only voiced by a small number of women in our feedback (4%) and one patient also commented that in hindsight she would have found regular hospital appointments ‘stressful’. This may be due to the fact that patients who were suitable for patient-initiated follow-up, were presented with it part of routine standard of care, hence unable to compare against the potential option of reassurance examinations.

It is also possible that patients may experience alarming symptoms and not self-present. There are barriers to accessing services, such as lack of confidence, was reflected in written feedback. However, 20% of patients discharged to self-initiated follow-up celled with symptoms or concerns and were filtered into fast-track clinic, reflecting appropriate engagement with the service. It is important to note that there are obstacles to self-presentation of red flag symptoms. Studies have identified features such as older age, lower socio-economic status, comorbidity and atypical symptoms, as risk factors for delayed presentation [16, 17].These were taken into account when selecting women for self-initiated follow-up in this cohort.

The British Gynaecological Cancer Society stratifies stage 1b grade 2 endometrial cancer as an intermediate risk of recurrence. Our total recurrence for all stage 1b patients was 14.3% but for low-risk stage 1b grade 1 patients the recurrence rate was 9% (*n* = 1/11). The British Gynaecological Cancer Society still advises that intermediate risk endometrial cancer can be offered self-initiated follow-up after three-month review or at any time during the first two years of hospital follow-up, provided they have completed primary treatment and have no ongoing active or maintenance treatment [19]. Our data supports the safety of patient-initiated follow-up, particularly those defined as low-risk of recurrence with 100% progression free and overall survival in the 1a grade 1 cohort.

Following European Society for Medical Oncology guidance, the total number of hospital-based follow-up appointments for our total cohort would be 1372 over five years. At an estimate of £80/consultation, this would have cost the national health service a total of £109,760. Hospital follow-up also has a burden to the patient. There is evidence to support patient-initiated follow-up can reduce financial cost to the patient, due to paying for petrol, parking or public transport as well as the inconvenience of taking time to travel or arranging time out of employment to attend consultations [20]. So far eleven hospital-based consultations have been generated from patient-initiated follow-up, costing approximately £880. There were twenty telephone consultations with oncology nurses at an estimate of £866.60 [21]. The cost to run the Upbeat health course is around £500 for two hours a week for a 10-week course, with three or four course’s a year depending on uptake. This is an overall saving of 96.5% so far, in addition to this the patient does not have the timely and financial cost of travelling to the hospital. The financial benefit of this approach is echoed throughout literature [20, 21].

## Conclusion

This service evaluation supports the claim that patient-initiated follow-up represents a safe alternative to the traditional hospital-based protocol. Our service has received positive feedback from patients and demonstrated appropriate patient engagement. There is a potential for additional services to be offered, such as those described in this paper, to encourage and promote a healthy lifestyle to improve quality of life and reduce cardiovascular risk following early endometrial cancer surgery. Future research could investigate the effect of weight loss on rates of endometrial cancer recurrence. Patient- initiated schemes provide considerable financial benefit to both the national health system and the patient. In times of increasing health costs, falling budgets and post-pandemic economic uncertainty, this will be warmly received.
